# Measurement and Region Identification in Deep Displacement of Slopes Based on Rod-Fiber Coupling Structure

**DOI:** 10.3390/s22103623

**Published:** 2022-05-10

**Authors:** Pengzhen Liu, Zhen Liu, Cuiying Zhou

**Affiliations:** 1 School of Civil Engineering, Sun Yat-sen University, Guangzhou 510275, China; liupzh6@mail2.sysu.edu.cn (P.L.); zhoucy@mail.sysu.edu.cn (C.Z.); 2 Guangdong Engineering Research Centre for Major Infrastructure Safety, Guangzhou 510275, China; 3 Research Center for Geotechnical Engineering and Information Technology, Sun Yat-sen University, No. 135 Xingang West Road, Guangzhou 510275, China

**Keywords:** slope body, deep displacement of slopes, rod-fiber coupling structure, sensing experiment

## Abstract

For measuring and region-identifying the deep displacement of slopes, a rod-fiber coupling structure based on optical time-domain reflection technology was designed. Accuracy of measurement and region identification in the deep displacement of slopes were studied by calibration experiment and model experiment. A rod-fiber coupling structure was able to calculate the variation and accurately identify the region of deep displacement of a slope compared with the measured downslide displacement of the slope model. The maximum measurement error of the deep displacement of the slope was 10.1%, the identification error of the displacement region was less than 4.4%, and the accuracy of the displacement-region identification of the rod-fiber coupling structure was 3.1 cm. Thus, the rod-fiber coupling structure based on optical time-domain reflection technology can be used for measuring and for region identification in the deep displacement of the slopes, and can provide a new method for the identification of the sliding surfaces of slopes.

## 1. Introduction

Slope-engineering projects are frequently conducted in the construction fields of transportation, water conservancy, hydropower, and mining. The deep displacement of slopes may be greatly challenged under the influence of unfavorable geological structures, rainfall, water storage, and engineering construction, which can lead to landslide disasters.

Measurement of the deep displacement and depth identification of the displacement region of slopes are important ways to predict and control landslide disasters. The measurement and region identification of the deep displacement of slopes is of great significance to the study of the evolution and development of slope deformation, and it is necessary to comprehend the overall phenomenon of the deformation of slopes. Rather than relying on the low precision and automatic measurement of traditional inclinometers [1,2,3], Pei et al. applied optical fiber-sensing technology to a traditional inclinometer to improve its measurement precision [4]; however, this method needs to use the measurement reference point to calculate the deep displacement and is still limited in terms of structural complexity and instability. To reduce the displacement measurement error of slope mass, Kai et al. [5] adhered Fiber Bragg Grating (FBG) to the outer surface of a polyvinyl chloride (PVC) pipe; however, the FBG was easily fractured and shed with an increase in displacement, and it was difficult to measure the large lateral displacement. In addition, Zhu et al. [6] developed a cost-effective displacement sensor for the deep-displacement measurement of the slope based on single-mode fiber; however, the major structure of the sensor was easily damaged. The deep-displacement measurement of slopes is crucial for prediction and early warning systems with regard to landslide disasters. Using a large quantity of measurement data for the deep displacement of slopes, Zhang et al. [7] observed that the displacement of a landslide mass has the characteristics of ladder evolution. Furthermore, Lei et al. [8] and Xu et al. [9] discussed the synergistic effects of deep displacement and the internal force of the slope. Moreover, factors such as rainfall and temperature may also influence variation in the deep displacement of slopes [10,11]. He et al. [12] studied the instability process of the bedding rock slope model with weak interlayer under different rainfall conditions. Gischig et al. [13] revealed the influence of temperature on the deep displacement and progressive failure of slopes. To address the problem of progressive failure deformation of slopes, El Bedoui et al. [14] combined deep-seated gravitational deformations, geological structure, and other factors to study the different stages of landslides. The continuous accumulation of the deep displacement of a slope can easily lead to the destruction of the reinforcement structure and a landslide [15]; thus, the identification of the location of deep-displacement regions and the sliding surfaces of slopes provides important data support for accurate assessment of the landslide volume and disaster potential, and for landslide prewarning [16]. Commonly used theoretical methods, such as limit analysis and limit equilibrium methods, have been widely applied for identifying sliding surface [17,18]. Moreover, an increasing number of studies have tended to directly identify the accurate location of the deep-displacement regions and sliding surfaces of slopes using actual monitoring methods [19]. Xu et al. [20] studied a method to identify the sliding surface of a slope using microseismic monitoring technology. Sun et al. [21] and Zhu et al. [22] accurately identified the sliding surface of the slope using long-term monitoring. Carter et al. [23] investigated a method for monitoring the surface displacement of a slope to predict the position and shape of a sliding surface. In recent years, the method of utilizing optical time-domain reflection technology to detect environmental characteristics through a single-mode fiber has been discussed in many papers. The main problems needing further study in association with this technology are the highly restricted installation conditions [24,25] and the easily damaged sensing structure [26]. The identification of the deep-displacement region of a slope plays an important role in identifying the sliding surface of a slope. In an actual slope, the identification of the deep-displacement region and the sliding surface also provides important data and indexes of slope reinforcement; thus, the reinforcement cost can be reduced to ensure the safety of the slope.

This paper addresses the existing issues in measuring and identifying the deep displacement of the slope. A measuring device for measuring and region identification of the deep displacement of slopes is proposed, coupling the single-mode fiber and high elastic rubber rod. Furthermore, the structure and measurement principle of the rod-fiber coupling structure are introduced. The feasibility, reliability and measurement accuracy of the rod-fiber coupling structure for measuring the deep displacement of a slope are verified through calibration and model experiments. Then, the variation law of deep displacement and the results of the displacement region identification of the slope are analyzed in the overall sliding process of the slope model. In future work, the rod-fiber coupling structure will be applied in actual slope engineering to measure and identify the region of the deep displacement of the slope, and the measurement validity of the rod-fiber coupling structure in actual engineering monitoring will be verified.

## 2. Research Contents and Methods

### 2.1. Structure Design

**Figure 1:**
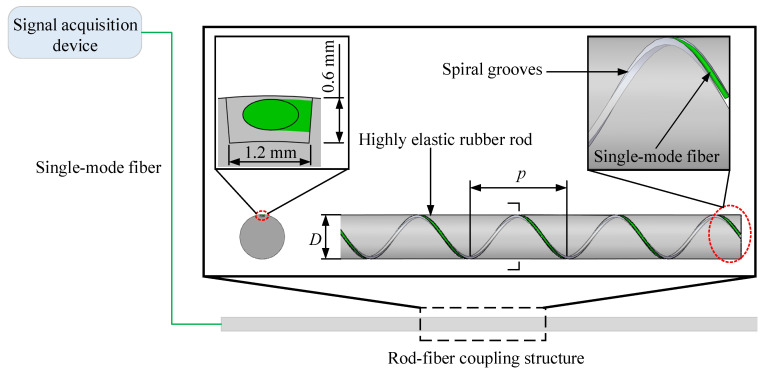
Structural diagram of rod-fiber coupling structure (*D* is the diameter of the highly elastic rubber rod, and *p* is the pitch of the spiral grooves).

To realize the measurement of deep displacement change throughout the process from the occurrence of deep displacement to the landslide of the slope, the device used for the measurement and region identification of the deep displacement of the slope primarily includes a signal acquisition device and rod-fiber coupling structure (Figure 1). The rod-fiber coupling structure bends under the extrusion of the slope mass, which further causes a change in the optical loss of the optical signal transmitted in the single-mode fiber. Additionally, the rod-fiber coupling structure indirectly obtains the variation information on the deep displacement of the slope.

As shown in Figure 1, the rod-fiber coupling structure can be divided into support structure and measurement structure. The support structure is a highly elastic rubber rod, and the measurement structure is a single-mode fiber. The main structure of the rod-fiber coupling structure is the highly elastic rubber rod. In addition, this study uses three-dimensional printing to manufacture spiral grooves on the surface of the highly elastic rubber rod. Spiral grooves with smooth inner surfaces make it easy for the single-mode fiber to couple with the highly elastic rubber rod. Spiral grooves improve the sensitivity of the single-mode fiber, the ability to measure the deep displacement and the accuracy of the displacement region identification, and effectively avoid the direct compression of slope mass to single-mode fiber along the diameter of the highly elastic rubber rod.

Backward Rayleigh scattering is the theoretical basis for the displacement measurement of the rod-fiber coupling structure. The single-mode fiber uniformly laid in the spiral grooves forms a continuous displacement measurement region on the highly elastic rubber rod. Furthermore, the backward Rayleigh scattering signal contains information on the optical loss value and location. When deep displacement of a slope occurs, the rod-fiber coupling structure and slope mass deform synchronously, which causes a change in the optical loss of the rod-fiber coupling structure. Therefore, the information on the deep displacement of the slope is transformed into a change in the optical loss. This information contains the corresponding relationship between the optical loss and the deep displacement of the slope, which is helpful in determining the deformation state and realizing the measurement and region identification of the deep displacement of the slope.

### 2.2. Measurement and Identification Principle

**Figure 2:**
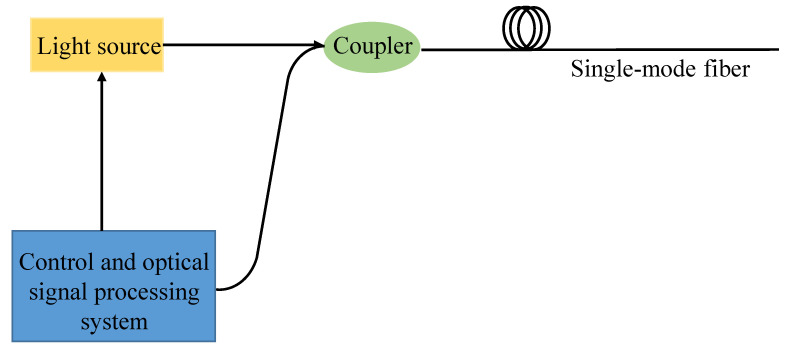
Schematic diagram of the signal acquisition device.

The signal acquisition device primarily comprises a light source and control and optical signal processing system. The backward Rayleigh scattering signal in the rod-fiber coupling structure is collected and analyzed by the signal acquisition device based on optical time-domain reflection technology. The schematic diagram of the signal acquisition device is shown in Figure 2. In the experiment, the signal acquisition device adopts the AE3000A optical time-domain reflectometer (OTDR) produced by Tianjin Deviser Instrument & Equipment Co., Ltd. (Tianjin, China). The OTDR can measure the transmission loss of single-mode optical fiber at 1310 nm and 1550 nm with a dynamic range up to 45 dB, and the maximum measuring range is 400 km. The minimum optical pulse is 3 ns, the loss threshold is 0.001 dB, and the minimum sampling resolution is 4 cm.

#### 2.2.1. Deep-Displacement Measurement Principle of Slope

**Figure 3:**
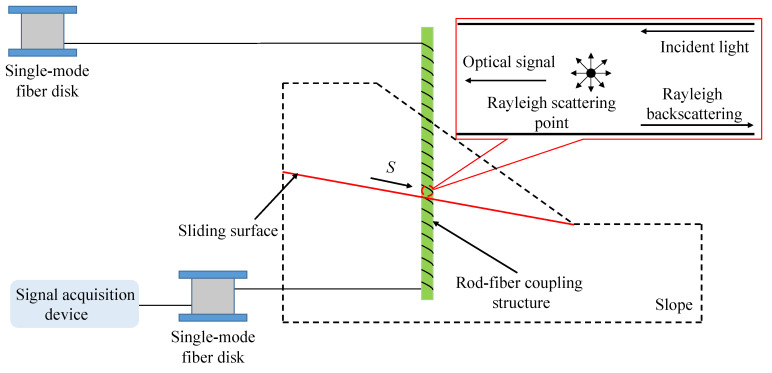
Measurement principle of deep displacement of slopes.

When a deep displacement occurs in the slope, the rod-fiber coupling structure laid in the slope bends; the deep-displacement measurement principle of the slope is shown in Figure 3. The slope mass and rod-fiber coupling structure below the sliding surface are fixed, and the friction resistance between the slope mass and the rod-fiber coupling structure is great [27]. Therefore, the deep displacement of slope model is transformed into the bending of the rod-fiber coupling structure in the process of slope model sliding. Furthermore, the bending of the rod-fiber coupling structure forces the optical signal transmitted in the single-mode fiber to generate optical loss, of which the Rayleigh scattering loss is the most significant [28]. When Rayleigh scattering occurs, a part of the incident light signal diffuses back along the axial direction of the single-mode fiber, a phenomenon termed backward Rayleigh scattering. The backward Rayleigh scattering light has a particular time delay and carries the optical loss information of the single-mode fiber. The optical power of the backward Rayleigh scattering is proportional to the incident optical power of the Rayleigh scattering point [29]. Therefore, the optical loss information at the Rayleigh scattering point can be obtained by measuring the backward Rayleigh scattering optical power, and the optical loss information can be used to measure the deep displacement of the slope.

#### 2.2.2. Deep-Displacement Region Identification Principle for Slopes

The signal acquisition device measures the optical loss and event point of a single-mode fiber via the backward Rayleigh scattering of the optical signal. According to the time-delay characteristics of the backward Rayleigh scattering, the backward Rayleigh scattering of the optical signal at different positions of the rod-fiber coupling structure, caused by the bending of the slope mass, returns to the signal acquisition device in sequence. The optical power attenuation curve and displacement region along the length of the rod-fiber coupling structure can be obtained by measuring the optical loss and transmission time of the received backward Rayleigh scattering of the optical signal [30]. In [30], we mainly studied the measuring sensitivity, axial resolution, and directional sensitivity of rod-fiber coupling structure. However, some problems, such as displacement-region identification, still need to be studied. According to the results of the present study, the structure of the highly elastic rubber rod and the measurement method of optical loss were improved. Thus, the stability and application range of the rod-fiber coupling structure were improved, and the distribution information of the deep-displacement region of the slope was able to be identified. Using the transmission speed of the optical signal in single-mode fiber and the time interval of the optical signal from launch to return, the representative equation of the distance *d* between the Rayleigh scattering point and the incident point of the optical signal can be expressed as [31]:

*d* = (*c* × *t*)/2*n*, (1)

where *t* is the time interval of the optical signal from launch to return, *c* is the speed of light.

### 2.3. Calibration Experiment Scheme

**Figure 4:**
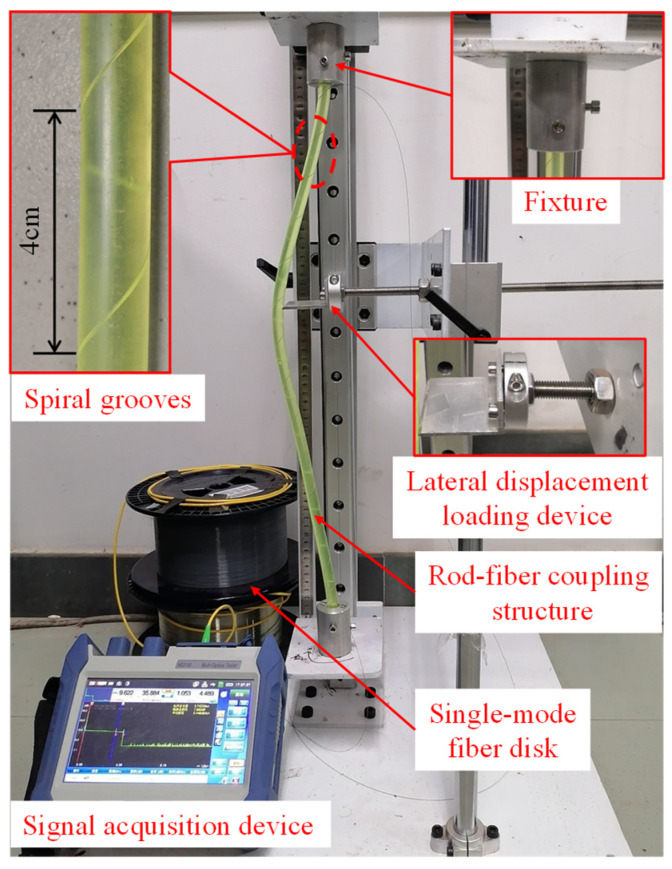
Scheme of the calibration experiment.

In the calibration experiment, the length and diameter of the rod-fiber coupling structure were 60 cm and 10 mm, respectively. The pitch, width, and depth of the spiral grooves were 4 cm, 1.2 mm, and 0.6 mm, respectively. The mechanical properties the highly elastic rubber rod at normal temperature, such as tensile strength, elongation at break, and shore hardness were 6.31 Mpa, 310%, and 77 HA, respectively. Furthermore, the two ends of the rod-fiber coupling structure were fixed during the lateral displacement loading. The scheme for the calibration experiment is shown in Figure 4. A lateral displacement with a step length of 0.5 cm was loaded slowly, and the maximum lateral loading displacement was 8.5 cm. The lateral displacement loading system of the rod-fiber coupling structure mainly adopts 304 stainless steel, and the fixture and lateral displacement loading device adopt aluminum alloy. Many experiments were carried out to select the method for fixing the single-mode fiber onto the surface of the highly elastic rubber rod. When cyanoacrylate glue was used to fix the single-mode fiber on the surface of the rubber completely, the single-mode fiber was easily broken during the strong bending action of the highly elastic rubber rod. In order to avoid the breakage of the single-mode fiber during the large bending of the rod-fiber coupling structure, the single-mode fiber was encapsulated in the spiral grooves by the transparent acrylic pressure sensitive adhesive tape. The bare silica single-mode fiber (G.652D) with acrylic resin coating was used in rod-fiber coupling structure in this paper.

Calibration was used to support the measurement experiment of deep displacement of the slope. The primary purpose of calibration is to obtain the optical loss of rod-fiber coupling structure when the calibration conditions are consistent with the deep displacement of slope measurement conditions from deformation caused by displacement. In accordance with this purpose, the main content of calibration is the response of optical loss of the rod-fiber coupling structure to the lateral displacement. The main influencing factors of calibration are the bending deformation and the axial stretching deformation of the rod-fiber coupling structure. According to the existing research results, the influencing factor of axial stretching deformation can be ignored; this is discussed in detail in Section 3.2. Furthermore, the influencing factor of bending deformation was fully considered in the calibration process. Therefore, the corresponding relationship between the lateral displacement and the optical loss of the rod-fiber coupling structure obtained by the calibration is effective and reliable for measuring the deep displacement of a slope.

### 2.4. Model Experiment Scheme

#### 2.4.1. Slope Model Design and Rod-Fiber Coupling Structures Installation

**Figure 5:**
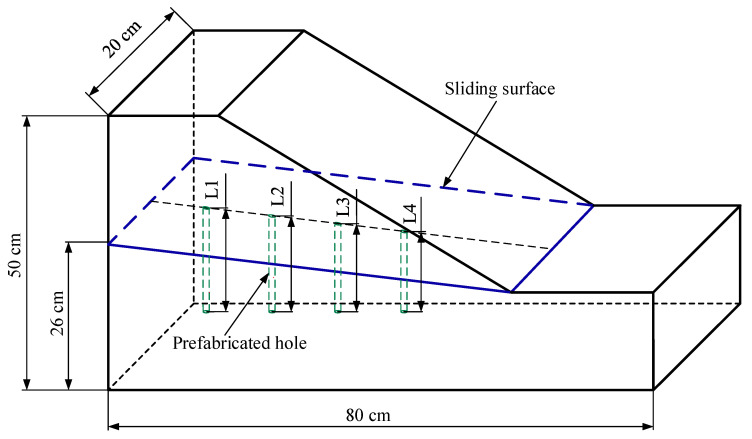
Schematic of slope model.

A slope model with a length of 80 cm, width of 20 cm, and height of 50 cm was used in this study to verify the effectiveness of the rod-fiber coupling structure in the measurement and region identification of the deep displacement of a slope, as shown in Figure 5. A sliding surface with an inclination angle of 10° was set 26 cm from the bottom of the slope. The sliding surface and supporting structure consisted of steel plates simulating a stable layer. The displacement of different positions on the slope can be used to understand the variation law of the deep displacement of the slope more comprehensively; therefore, in this study, four prefabricated holes were set on the sliding surface. To make different rod-fiber coupling structures subject to the same load, the four prefabricated holes for installing the rod-fiber coupling structure were set on the central line of the sliding surface. Moreover, the axial spacing between the prefabricated holes was set at 12 cm to avoid possible interaction between adjacent rod-fiber coupling structures [32]. Furthermore, the diameter of the prefabricated holes was 1.2 cm, and the depths L1, L2, L3, and L4 of prefabricated holes I, II, III, and IV were 16 cm, 13.9 cm, 11.8 cm, and 9.7 cm, respectively. Back-filling in layers was used to fill the model to obtain the state of a natural slope to the greatest extent possible. Furthermore, the completed slope model was timed to start a period before the model experiment.

**Figure 6:**
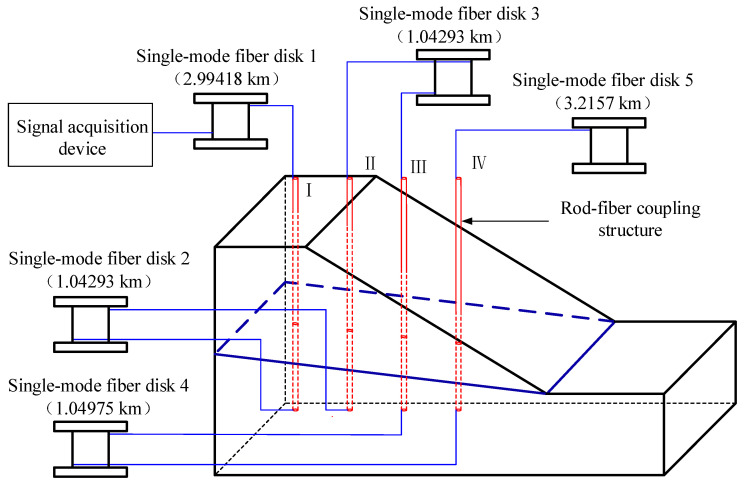
Installation of rod-fiber coupling structures.

Four rod-fiber coupling structures were fixed in the prefabricated holes, connected in series on an optical fiber line, and finally connected to the signal acquisition device through the single-mode fiber. To avoid the influence of the dead zone and Fresnel reflection, which exist in any measurement that uses a signal acquisition device [33], five single-mode fiber disks of a particular length were arranged in the optical fiber line. The installation of rod-fiber coupling structures is shown in Figure 6.

#### 2.4.2. Slope Model Building and Lateral Loading

**Figure 7:**
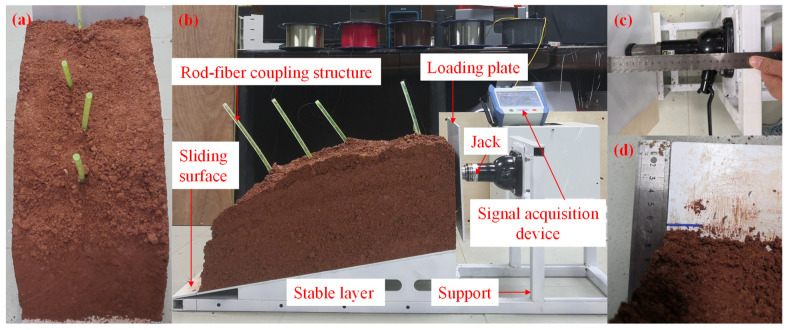
Schematic of slope model experiment. (**a**) rod-fiber coupling structures installation; (**b**) slope model load; (**c**) lateral loading displacement measurement; (**d**) slope toe downslide displacement measurement.

A jack was used to laterally load the slope model to simulate the sliding process of the slope mass. The slope mass located in the upper part of the stable layer slid down along the sliding surface under lateral loading. During the process of slope model building, the rod-fiber coupling structures were fixed in the prefabricated holes before filling the soil, as shown in Figure 7a. The lateral loading system of the slope model primarily comprised a support, jack, and loading plate. The loading plate was square with a side length of 20 cm. The jack is shown in Figure 7b Its maximum load and stroke were 2 tons and 20 cm, respectively. The lateral loading displacement was measured with a steel ruler during the experiment to ensure accuracy, as shown in Figure 7c. The downslide displacement of the slope toe was simultaneously measured to obtain the actual displacement of the slope model during the lateral loading process, as shown in Figure 7d.

The displacement variation of a deep slope can be simulated during slope-mass sliding using a model experiment, which further verifies the feasibility and effectiveness of the rod-fiber coupling structure for the measurement and region identification of the deep displacement of the slope. It also provides theoretical support for the design of a more appropriate deep-displacement measurement device for slopes.

## 3. Results and Discussion

### 3.1. Influence of Pitch and Diameter on Initial Optical Loss of Rod-Fiber Coupling Structure

**Figure 8:**
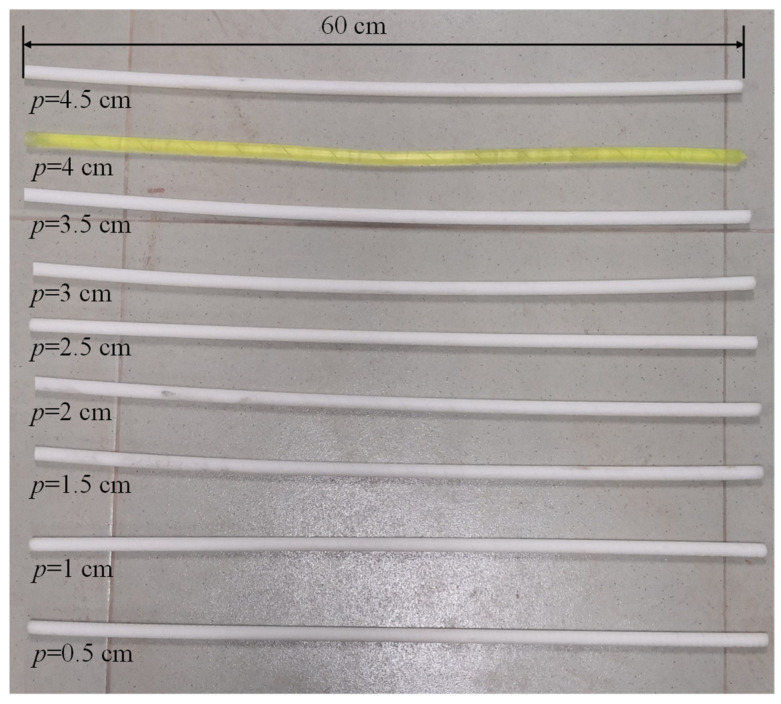
Highly elastic rubber rods of different pitches with a diameter of 10 mm and a length of 60 cm.

**Figure 9:**
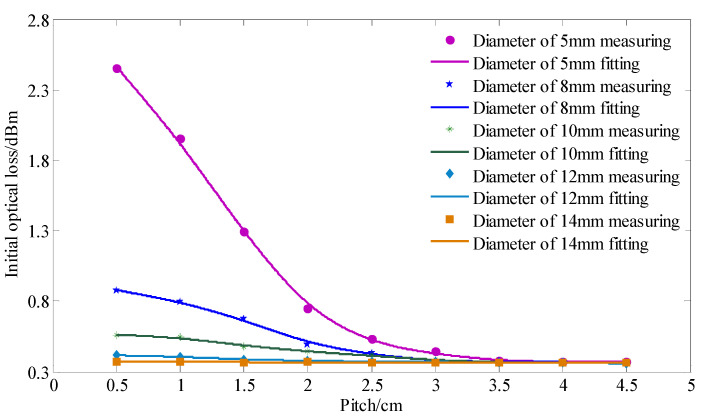
Influence of pitch and diameter on initial optical loss of rod-fiber coupling structure.

Aiming at the effects of various factors on the optical loss measuring, the arithmetic mean method was used to eliminate the influence of the fluctuation of the optical loss obtained by the signal acquisition device. To study the influence of pitch and diameter on the initial optical loss of the rod-fiber coupling structure, rod-fiber coupling structures with diameters of 5 mm, 8 mm, 10 mm, 12 mm, and 14 mm, and pitches of 0.5 cm, 1 cm, 1.5 cm, 2 cm, 2.5 cm, 3 cm, 3.5 cm, 4 cm, and 4.5 cm were selected for the experiment. The highly elastic rubber rods of different pitches with a diameter of 10 mm and a length of 60 cm are shown in Figure 8. When the number of winding turns was one, the initial optical loss of the rod-fiber coupling structure was obtained, as shown in Figure 9. As observed in Figure 9, an increase in pitch caused the initial optical loss of the rod-fiber coupling structure to attenuate continuously; however, the attenuation slowed. For the same pitch and winding turns, the smaller the spiral winding diameter, the greater the initial optical loss of the rod-fiber coupling structure, and the greater the increasing range of the initial optical loss. With the increase in pitch, the fluctuation range of initial optical loss of rod-fiber coupling structures with different diameters decreased gradually. Furthermore, the initial optical loss could limit the length and measurement range of the rod-fiber coupling structure.

### 3.2. Fitting Curve for Lateral Loading Displacement and Optical Loss

According to the bending loss principle of single-mode fiber, the optical loss of the spiral winding of single-mode fiber decreases with the increase in diameter and pitch [34]. The axial stretching sensitivity of the spiral winding of single-mode fiber decreases with the increase in diameter (from 5.2 mm to 6 mm) and pitch (from 1.2 cm to 1.3 cm), and the axial stretching deformation has little influence on the optical loss changing of the spiral winding of single-mode fiber after the radius of curvature exceeds a certain value [35]. Therefore, the axial stretching deformation of the rod-fiber coupling structure with a diameter of 10 mm and pitch of 4 cm had no obvious influence on its optical loss change. The optical loss was due mainly to the bending of the rod-fiber coupling structure in the calibration experiment. Furthermore, there was a corresponding excellent relation between the lateral loading displacement and the optical loss of the rod-fiber coupling structure. The corresponding relation can be established by numerical fitting, whose input parameter is the optical loss of rod-fiber coupling structure, and the output parameter is lateral loading displacement. The optical loss of the rod-fiber coupling structure obtained from the calibration experiment and the lateral loading displacement was numerically fitted, and primary, secondary, and cubic fittings were performed. The fitting equation is expressed as follows:

y = a_1_x^3^ + a_2_x^2^ + a_3_x + b, (2)

where y is the lateral loading displacement, and x is the optical loss of the rod-fiber coupling structure.

**Figure 10:**
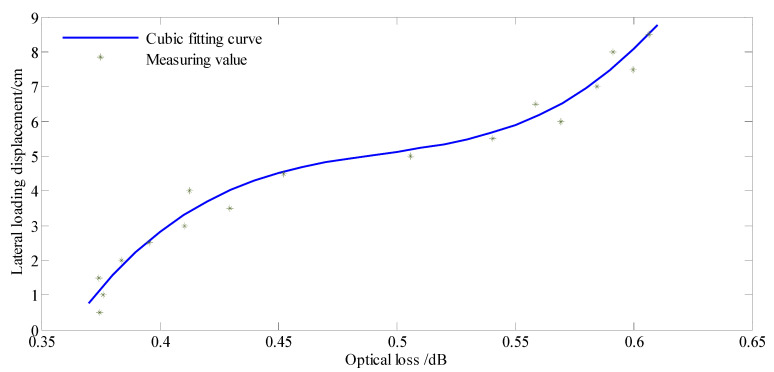
Cubic fitting curve for lateral loading displacement and optical loss.

The fitting curve parameters for lateral loading displacement and optical loss are listed in Table 1. As observed in the table, the regression coefficient of the cubic fitting equation was the highest. Therefore, the cubic fitting equation was selected to calculate the lateral displacement of the rod-fiber coupling structure in the model experiment. The cubic fitting curve, with optical loss as the independent variable and lateral loading displacement as the dependent variable, is shown in Figure 10. In the figure, the optical loss positively correlates with the lateral displacement of the rod-fiber coupling structure.

### 3.3. Analysis of Measurements of Deep Displacement of Slopes

#### 3.3.1. Measurement Error of Rod-Fiber Coupling Structure

**Figure 11:**
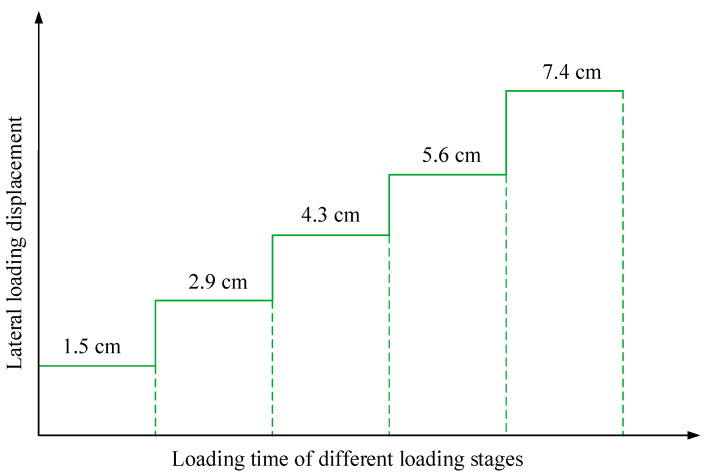
Lateral loading displacement.

**Figure 12:**
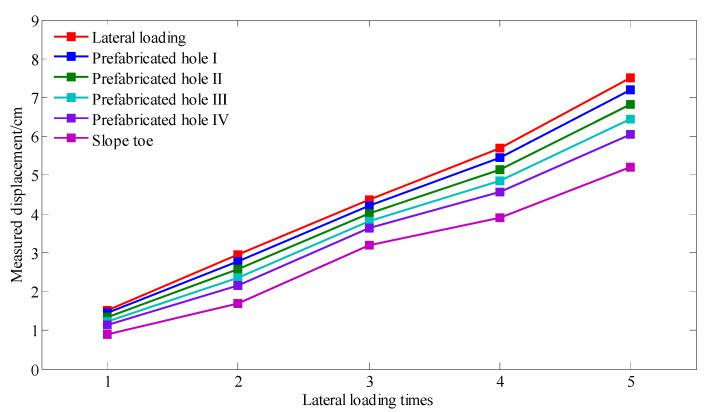
Measurements of the displacement of lateral loading, downslide displacement of the slope toe, and displacement of the slope mass at the four prefabricated holes.

During the model experiment, the loading plate compressed the side of the slope model, thereby resulting in an increase in the downslide strength and overall downward movement of the slope model. Lateral loading was applied in stages, as shown in Figure 11. The soil was compressible [36]; therefore, the downslide displacement of the slope toe was smaller than the lateral loading displacement. We consider that the experimental model was small, the downslide displacement was short, and the slope model was only affected by the friction of the sliding surface. Therefore, the compression of the soil can be considered to be an approximate linear variation during the lateral loading process. The measured values of the displacement of the lateral loading, downslide displacement of the slope toe, and displacement of the slope mass at the four prefabricated holes are shown in Figure 12. The displacement variation curve in the figure shows that the displacement of different parts of the slope model was different under the same lateral loading.

**Figure 13:**
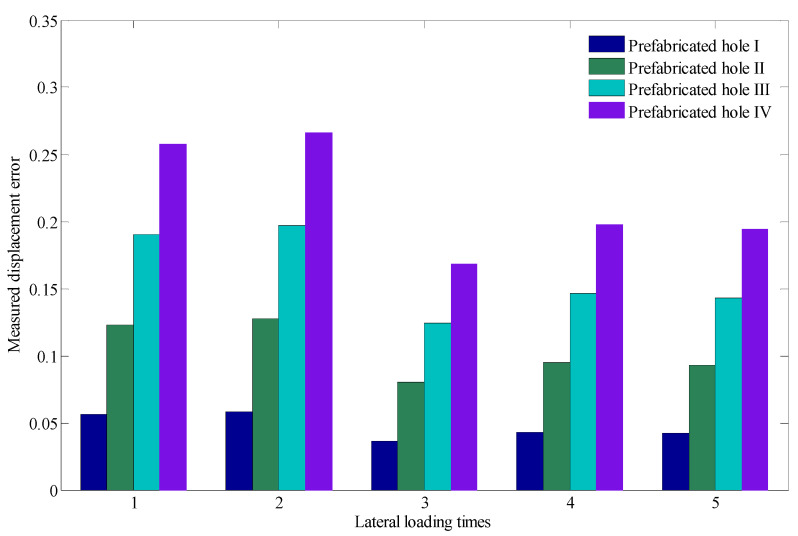
Measurement errors between the lateral loading displacement and displacement of the slope mass at the four prefabricated holes.

To further study the displacement of the slope mass at the four prefabricated holes under the same lateral loading displacement, the measurement errors between the displacement of lateral loading and displacement of the slope mass at the four prefabricated holes were analyzed, as shown in Figure 13. Due to the deformation characteristics of the slope mass, the measurement displacement error was positively associated with the distance between slope mass and loading plate. Furthermore, with the increase in lateral loading displacement, the measurement displacement error showed a general decreasing trend.

**Figure 14:**
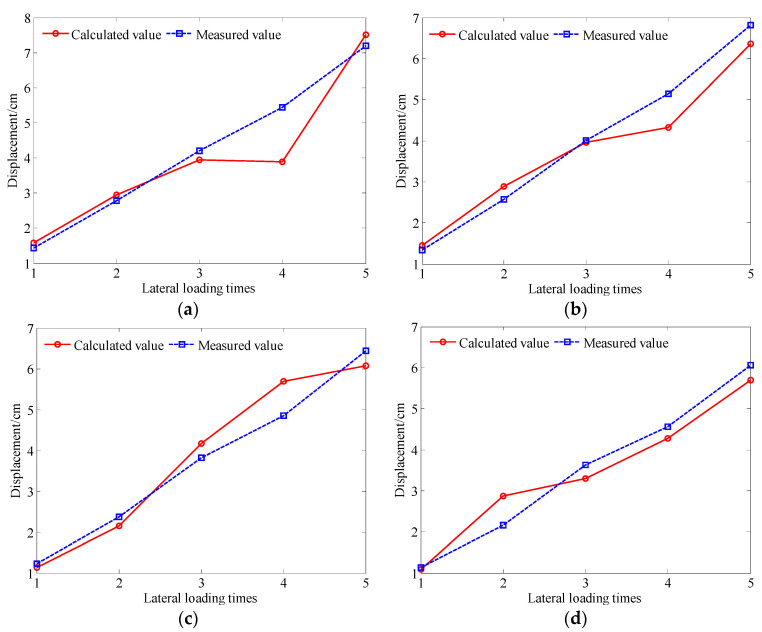
Calculated and measured deep displacement of a slope under different lateral loading times: (**a**) rod-fiber coupling structure I; (**b**) rod-fiber coupling structure II; (**c**) rod-fiber coupling structure III; (**d**) rod-fiber coupling structure IV.

The optical loss produced by the different degrees of bending of the rod-fiber coupling structures I, II, III, and IV are measured by the signal acquisition device during slope sliding process, and the deep displacement of the slope is calculated according to Equation (2). The calculation results for the displacement of the different rod-fiber coupling structures and the measured displacement of the slope mass at the four prefabricated holes under the different lateral loading displacements are shown in Figure 14. As observed in the figure, the difference between the calculated and measured displacements was small, and the maximum error was 10.1%. A rod-fiber coupling structure with a smaller measurement error will be researched in future work to achieve measurement requirements of deep displacement of slopes [37]. The experimental results show that the rod-fiber coupling structure offers a high-accuracy measurement of the deep displacement of the slope.

#### 3.3.2. Difference Analysis of the Measurement Characteristics of Different Rod-Fiber Coupling Structures

**Figure 15:**
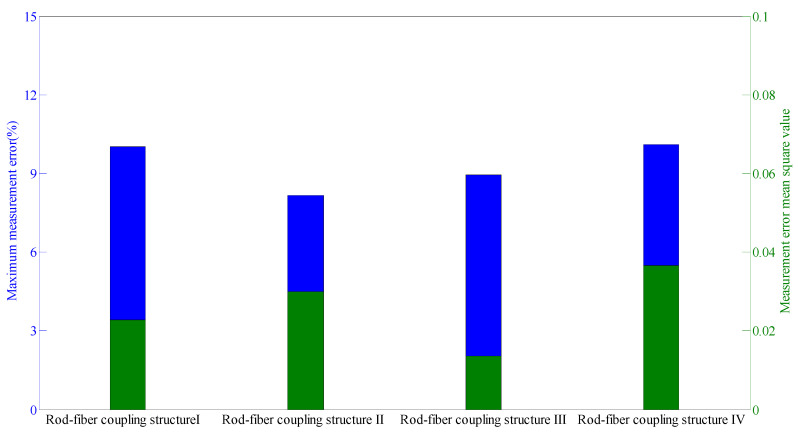
Measurement error mean square value and maximum measurement error of rod-fiber coupling structures I, II, III, and IV.

The measurement errors were analyzed during the lateral displacement loading to study the measurement characteristics of rod-fiber coupling structure, as shown in Figure 15. The measurement error mean square value of rod-fiber coupling structure III was the smallest indicating that rod-fiber coupling structure III had good measurement characteristics, its maximum measurement error being approximately 9%. Thus, the rod-fiber coupling structure exhibits good stability in the deep-displacement measurement of a slope. Furthermore, the measurement accuracy of the rod-fiber coupling structure can be effectively improved by controlling fabrication accuracy.

### 3.4. Analysis of Displacement-Region Identification Error

**Figure 16:**
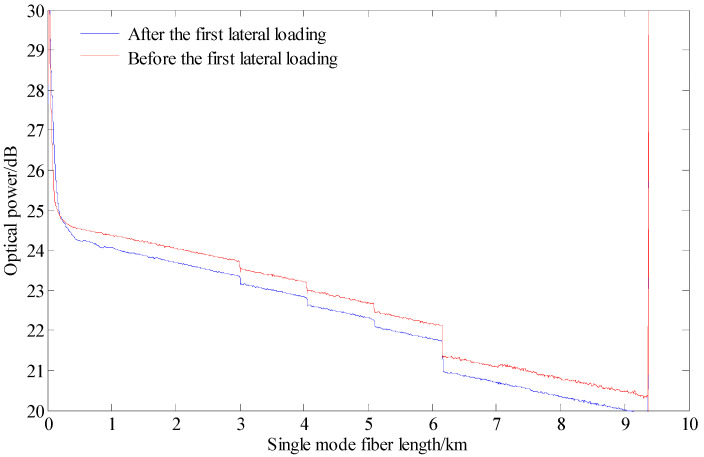
Events distribution of the single-mode fiber line before and after the first lateral loading.

In order to compare and analyze the change of optical loss of the rod-fiber coupling structure before and after lateral loading, the events and optical power distribution of the single-mode fiber line before and after the first lateral loading are shown in Figure 16. Before the first lateral loading, it could be observed that there were four events in the single-mode fiber line within the range of 2.5–6.5 km due to the initial optical loss of rod-fiber coupling structures. After the first lateral loading, the optical loss of rod-fiber coupling structures increased on the basis of initial optical loss. Using the distribution of the rod-fiber coupling structures in the slope model, it can be speculated that the above events before the first lateral loading were backward Rayleigh scattering points caused by spiral winding of single-mode fiber. After the first lateral loading, the above events were backward Rayleigh scattering points caused by the bending of rod-fiber coupling structure.

**Figure 17:**
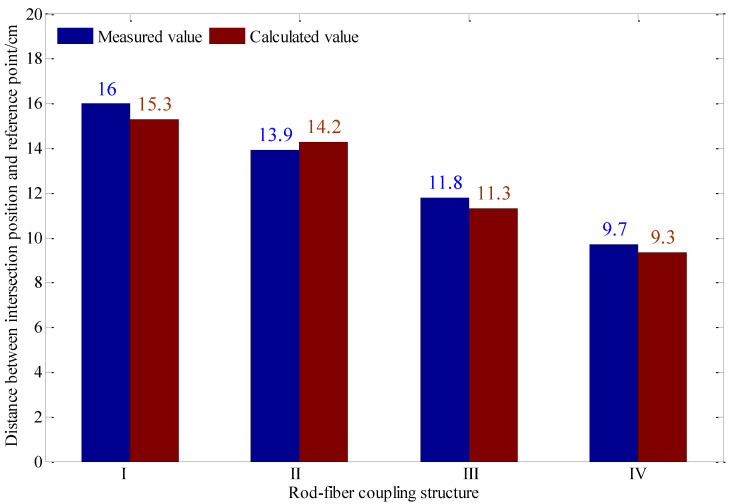
The distance between the intersection position of the rod-fiber coupling structure and sliding surface and the reference point.

The intersection of the rod-fiber coupling structure and the sliding surface deformed in the process of slope mass sliding along the sliding surface. The distance between the intersection position of the rod-fiber coupling structure and the sliding surface and the lower endpoint of rod-fiber coupling structure is reported in Figure 6. The displacement region can be identified by taking the lower endpoint of rod-fiber coupling structure as the reference point. The distance between the intersection position of the rod-fiber coupling structure and sliding surface and the reference point is shown in Figure 17. The maximum error of identifying the intersection position using the principle of displacement-region identification was 4.4%. The event measured by the signal acquisition device was the backward Rayleigh scattering information of a point on the rod-fiber coupling structure. The bending of the rod-fiber coupling structure was a curve of a particular length. Therefore, there was a particular error in locating the intersection position of the rod-fiber coupling structure and sliding surface using the principle of displacement-region identification.

**Figure 18:**
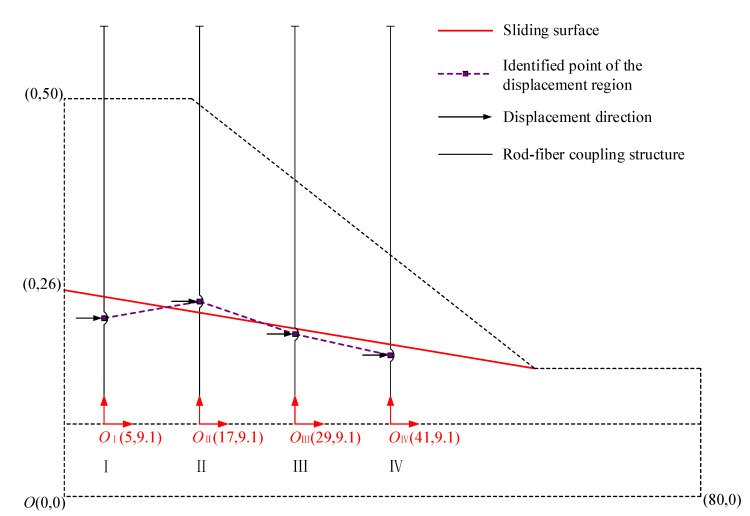
Sliding surface according to the identified point of the displacement region (unit: cm).

The distribution of the identified point of the displacement region on the rod-fiber coupling structure was studied to further analyze the feasibility of identifying the sliding surface of the slope according to the principle of displacement region identification. Figure 18 shows that the sliding surface was very close to the actual sliding surface, according to the identified point of the displacement region. Therefore, it can be concluded that the rod-fiber coupling structure had good application potential for the identification of sliding surface.

Based on the above identification error analysis, the variation law of the deep displacement slopes and the location of the displacement region in the overall sliding process of the slope model can be obtained by reasonably installing the rod-fiber coupling structure, which provides a theoretical basis for evaluating the slope stability and degree of possible disaster. In the model experiment, the rod-fiber coupling structure was mainly used to measure the lateral displacement of slope mass. Considering a measurement device with similar functions [38], the maximum measurement of lateral displacement with a rod-fiber coupling structure with a diameter of 10 mm and pitch of 4 cm was increased from 3.6 cm to 7.4 cm. According to the expansion equation of the cylindrical helix [39], the axial resolution of the rod-fiber coupling structure with a diameter of 10 mm and pitch of 4 cm q_1_ can be defined as:

(3) $$q_{1}=\frac{q_{0}p}{\sqrt{{\left(\pi D\right)}^{2}+p^{2}}},$$

where *p* is the pitch, *D* is the diameter, and *q*_0_ is the minimum sampling resolution of the OTDR.

After calculation, the accuracy of the displacement-region identification of the rod-fiber coupling structure was 3.1 cm. Therefore, the displacement-region identification accuracy was increased from 4 cm to 3.1 cm, showing that the rod-fiber coupling structure has good application prospects for slope safety monitoring.

### 3.5. Application Analysis of the Rod-Fiber Coupling Structure in Actual Slope Engineering

The pitch and curvature of the spiral winding of the single-mode fiber on the highly elastic rubber rod are constant. Therefore, when the same displacement occurs at different positions of the rod-fiber coupling structure, a similar corresponding relation between displacement and optical loss can be obtained. In actual deep lateral displacement measurement, the rod-fiber coupling structure can be customized to a specific length by splicing the two ends of the highly elastic rubber rod according to the engineering requirements. Furthermore, the cubic fitting equation parameters must be recalibrated before installation of the new rod-fiber coupling structure. Many related problems remain to be studied before practical application of the rod-fiber coupling structure; these include encapsulation, tensile test, and full-scale lateral displacement test.

The rod-fiber coupling structure and cubic fitting equation with regard to optical loss and lateral loading displacement can be applied to the deep-displacement monitoring of actual slopes, and a special encapsulation structure for the rod-fiber coupling structure is in development. In actual slope engineering, the rod-fiber coupling structure must pass through the potential sliding surface of the slope during the installation process. The lower end of the rod-fiber coupling structure should be fixed in the stable layer to measure the deep displacement and identify the displacement region of the slope more accurately. The rod-fiber coupling structure should avoid extensive bending, and an initial optical loss as much as possible during the installation process. A rod-fiber coupling structure with a simple structure, high working efficiency, and low cost can be used to construct a large displacement monitoring system for large slope engineering by connecting the structures in series. The rod-fiber coupling structure can be used to construct a monitoring system for actual slope engineering. Based on the information on deep displacement and the position of the displacement region, the health of actual slopes can be monitored, and landslide disaster early-warning methods can be proposed.

## 4. Conclusions

In this study, a rod-fiber coupling structure was proposed for deep-displacement measurement, displacement-region identification and sliding surface identification in a slope. The feasibility and measurement error of the rod-fiber coupling structure were analyzed through calibration and model experiments. The major findings were as follows:

The application of a rod-fiber coupling structure based on optical time-domain reflection technology in the deep-displacement measurement of the slope effectively improves the applicability and measurement accuracy of optical fiber sensing technology in deep-displacement measurement and displacement-region identification of the slope.

A decrease in pitch and diameter significantly increases the initial optical loss of the rod-fiber coupling structure. The maximum error of the rod-fiber coupling structure in the deep-displacement measurement was 10.1%, the error in the displacement-region identification was less than 4.4%, and the displacement-region identification accuracy was increased from 4 cm to 3.1 cm.

A rod-fiber coupling structure with a simple structure and high signal acquisition automation provides a new method for the construction of a remote deep-displacement monitoring system for large complex slopes and for the identification of sliding surfaces.

## Figures and Tables

**Table 1:** Parameters of fitting curve for lateral loading displacement and optical loss.

Fitting Equation	a_1_	a_2_	a_3_	b	Regression Coefficient
Primary	/	/	26.66	−8.307	0.9362
Secondary	/	−31.21	57.07	−15.47	0.9392
Cubic	1652	−2448	1218	−198.5	0.9781

## Data Availability

The data are presented in the paper.
